# Sulfur Compounds Inhibit High Glucose-Induced Inflammation by Regulating NF-κB Signaling in Human Monocytes

**DOI:** 10.3390/molecules25102342

**Published:** 2020-05-17

**Authors:** Eun Seong Jo, Nipin Sp, Dong Young Kang, Alexis Rugamba, Il Ho Kim, Se Won Bae, Qing Liu, Kyoung-Jin Jang, Young Mok Yang

**Affiliations:** 1 Department of Pathology, School of Medicine, Institute of Biomedical Science and Technology, Konkuk University, Chungju 27478, Korea; eses0706@naver.com (E.S.J.); nipinsp@gmail.com (N.S.); kdy6459@naver.com (D.Y.K.); rugambalex@gmail.com (A.R.); 2 Nara Bio Co., Ltd., Jeonju, North Jeolla 54852, Korea; 79421515@naver.com; 3 Green Chemistry & Materials Group, Korea Institute of Industrial Technology (KITECH), Cheonan 31056, Korea; swbae@kitech.re.kr; 4 Department of Green Process and System Engineering, Korea University of Science & Technology (UST), Cheonan 31056, Korea; 5 Jilin Green Food Engineering Research Institute, Changchun 130000, China; liuqing0523@hotmail.com

**Keywords:** High glucose, Diabetes, TLRs, NF-κB, Canonical pathway, PKC pathway, Proinflammatory cytokines

## Abstract

High glucose-induced inflammation leads to atherosclerosis, which is considered a major cause of death in type 1 and type 2 diabetic patients. Nuclear factor-kappa B (NF-κB) plays a central role in high glucose-induced inflammation and is activated through toll-like receptors (TLRs) as well as canonical and protein kinase C-dependent (PKC) pathways. Non-toxic sulfur (NTS) and methylsulfonylmethane (MSM) are two sulfur-containing natural compounds that can induce anti-inflammation. Using Western blotting, real-time polymerase chain reaction, and flow cytometry, we found that high glucose-induced inflammation occurs through activation of TLRs. An effect of NTS and MSM on canonical and PKC-dependent NF-κB pathways was also demonstrated by western blotting. The effects of proinflammatory cytokines were investigated using a chromatin immunoprecipitation assay and enzyme-linked immunosorbent assay. Our results showed inhibition of the glucose-induced expression of TLR2 and TLR4 by NTS and MSM. These sulfur compounds also inhibited NF-κB activity through reactive oxygen species (ROS)-mediated canonical and PKC-dependent pathways. Finally, NTS and MSM inhibited the high glucose-induced expression of interleukin (IL)-1β, IL-6, and tumor necrosis factor-α and binding of NF-κB protein to the DNA of proinflammatory cytokines. Together, these results suggest that NTS and MSM may be potential drug candidates for anti-inflammation therapy.

## 1. Introduction

Atherosclerosis is a disease caused by the deposition of plaques of fatty substances on the inner walls of arteries and is considered the main factor contributing to death in type 1 and type 2 diabetes [1,2]. Glucose is the key factor that induces these conditions, in which high glucose may lead to the accumulation of diacylglycerol and the activation of protein kinase C (PKC) in vascular cells, thereby upregulating the glucose flux via the aldose reductase pathway [3]. Studies have shown that these pathways are connected with inflammation through nuclear factor-kappa B (NF-κB) activation [4,5]. In atherosclerosis, high glucose conditions activate NF-κB, which then activates various inflammatory genes, including adhesion molecules that promote the adhesion of monocytes to endothelial cells [3]. 

NF-κB is a transcription factor that regulates immune responses and acts as a mediator of inflammatory responses [6]. As a result of inflammation, pathogen-associated molecular patterns and inflammatory cytokines stimulate certain cell surface receptors, such as toll-like receptors (TLRs), to activate NF-κB and initiate inflammatory immune response signaling pathways [7]. TLRs identify molecular patterns in response to pathogens to induce innate immune responses [8,9]. The activation of TLRs triggers the signaling pathway to produce cytokines and initiate an immune response [10]. Studies have shown that TLR expression is upregulated in diabetes (type 1 diabetes) [11] and atherosclerosis [12]. Among TLRs, TLR2 and TLR4 are considered to play important roles in atherosclerosis as their expression is increased in this disease [13,14]. Myeloid differentiation factor 88 (MyD88) is a common adaptor for TLR2 and TLR4, and the loss of MyD88 leads to anti-inflammation as well as the downregulation of tumor necrosis factor-α (TNF-α) and members of the interleukin (IL)-1-receptor (IL-1R) family [15]. An immune response occurs as a result of hyperglycemia, inflammation, or diabetes. As a result, TLR2 and TLR4 expression increases with the increase in NF-κB expression according to inflammation responses [16].

The activation of NF-κB in response to inflammation involves canonical and non-canonical pathways. The canonical pathway responds to diverse stimuli that activate different cytokine receptors [17], whereas the non-canonical pathway responds to a specific group of stimuli and ligands of TNF receptor superfamily members [18,19]. In response to cell stimulation in the canonical pathway, NF-κB activation is triggered by a multi-subunit IκB kinase (IKK) complex, which is activated with the help of IκBα degradation [20]. The IKK complex consists of two catalytic subunits, IKKα and IKKβ, and a regulatory subunit, IKKγ. The IKK complex is considered as the master regulator of NF-κB-dependent inflammatory responses [21,22]. Among those, IKKβ is necessary for the activation of NF-κB [23], whereas IKKα plays a role in the development and differentiation of the epidermis [24].

TLRs play a vital role in inflammation by acting as receptors for the inflammatory responses to PKCα, which is normally involved in cell growth and differentiation. In hyperglycemic conditions, reactive oxygen species (ROS) are upregulated, and then the phosphorylation of PKCα induced by ROS mediates the release of IL-1β through the phosphorylation of ERK1/2 and p38 MAPK that leads to the activation of NF-κB [25,26]. Hyperglycemic conditions activate p38 MAPK [27], which then triggers the production of other inflammatory cytokines, such as TNF-α and IL-6, through NF-κB activation during inflammatory responses [28,29].

Sulfur is an essential element in cysteine and methionine amino acids, but in humans, it must be consumed indirectly in the form of food, such as garlic, onions, or duck meat [30]. To use naturally occurring mineral sulfur, different substances are mixed with precipitated mineral sulfur to remove the toxic components in it. Many countries, including the USA, use a non-toxic form of dietary sulfur or natural sulfur for the treatment of many diseases, including inflammatory diseases [31]. A recent study demonstrated that non-toxic sulfur (NTS) extracted and processed from mineral sulfur enhances growth hormone signaling in mouse muscle cells [32]. Methylsulfonylmethane (MSM) is also a natural organic sulfur-containing compound present in food and beverages [33]. MSM has various properties, such as anticancer [34], antioxidant [35], and anti-ketotic [36] activities, which make it a promising drug candidate. MSM also has the ability to inhibit inflammatory responses in relation to TNF-α in cardiac cells [37].

In the present study, the effects of NTS and MSM on inflammation due to high glucose were determined. To evaluate the anti-inflammatory effects of NTS and MSM, molecular analyses of TLR and NF-κB signaling pathways were also analyzed under high glucose conditions.

## 2. Results

### 2.1. Sulfur Compounds Inhibit the High Glucose-Induced Expression of TLRs

**Figure 1:**
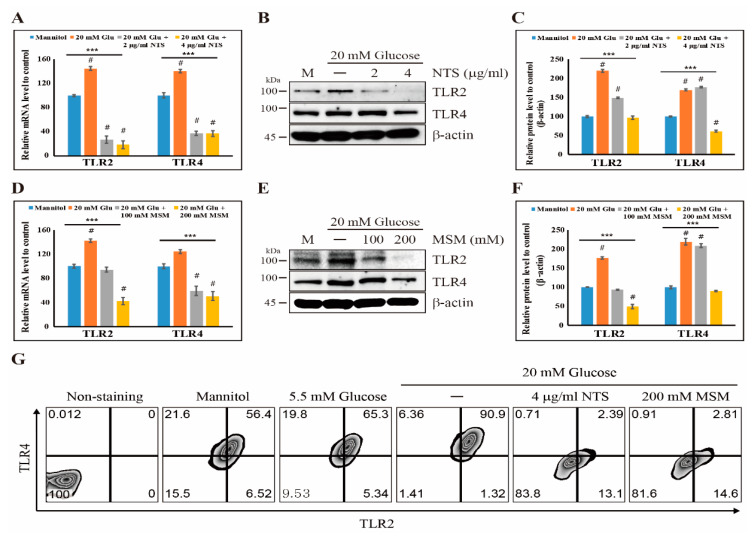
Sulfur compounds inhibit the expression of TLRs. (**A**) The expression levels of TLR2 and TLR4 mRNA in THP-1 cells were detected by real-time PCR after treatment with 20 mM glucose and NTS (2 and 4 μg/mL) for 24 h and normalized to GAPDH mRNA. The values are presented as means  ±  SEM of three independent experiments performed in duplicate (*n*  =  3). *** *p* < 0.001 (*ANOVA* test). # The mean difference is significant at the 0.001 level. (**B**) Western blot analysis of THP-1 cells treated with 2 and 4 µg/mL NTS for 24 h showing the inhibition of high glucose-induced TLR2 and TLR4 expression. (**C**) The representative expression levels of proteins following NTS treatment were determined by densitometry and normalized to β-actin. The values are presented as means  ±  SEM of three independent experiments performed in duplicate (*n*  =  3). *** *p* < 0.001 (*ANOVA* test). # The mean difference is significant at the 0.001 level. (**D**) Real-time PCR data of mRNA after treatment with 20 mM glucose and MSM (100 and 200 mM) for 24 h showing the relative expression levels of TLR2 and TLR4 normalized to GAPDH mRNA. The values are presented as means  ±  SEM of three independent experiments performed in duplicate (*n*  =  3). *** *p* < 0.001 (*ANOVA* test). # The mean difference is significant at the 0.001 level. (**E**) Western blotting analysis of THP-1 cells treated with 100 and 200 mM MSM for 24 h showing the inhibition of high glucose-induced TLR2 and TLR4 expression. (**F**) The representative expression levels of proteins following MSM treatment were determined by densitometry and normalized to β-actin. The values are presented as means ± SEM of three independent experiments performed in duplicate (*n*  =  3). *** *p* < 0.001 (*ANOVA* test). # The mean difference is significant at the 0.001 level. (**G**) Flow cytometry analysis showing the inhibition of the high glucose-induced expression of TLR2 and TLR4 by 4 µg/mL NTS and 200 mM MSM. Mannitol and 5.5 mM glucose were used as negative controls for high glucose-induced inflammatory reaction. Non-staining is the control not stained with anti-TLR2 and 4 antibodies for flow cytometry.

To evaluate the anti-inflammatory effects of sulfur compounds, THP-1 cells were treated with 2 and 4 μg/mL NTS and 100 and 200 mM MSM. First, we identified the highest concentration of glucose that induced inflammatory effects, and we found that the glucose concentration of 20 mM increased the expression of NF-κB, TLR4, p-IκBα, p-IKKα/β, and p-ERK in THP-1 cells (Figure S1). The cell viability assay was used to determine the optimal concentrations of two sulfur compounds with low cytotoxicity (Figure S2). Then, we analyzed the expression pattern of TLRs (TLR2 and TLR4) with two sulfur compounds in a high glucose condition. The results showed that 20 mM of glucose increased the mRNA expression of TLR2 and TLR4 compared with mannitol-treated control cells. However, these effects were decreased with 4 μg/mL NTS or 200 mM MSM (Figure 1A,D). We also analyzed the protein expression of TLR2 and TLR4 in the presence of NTS or MSM. These results confirmed the inhibition of the high glucose-induced expression of TLR2 and TLR4 by NTS and MSM (Figure 1B,C,E,F). Flow cytometry analysis of TLR2 and TLR4 confirmed the downregulation of TLRs in the surface of THP-1 cells by these sulfur compounds (Figure 1G). Furthermore, sulfur compounds effectively inhibited the protein expression of inflammatory NF-κB and TLR4 like a TLR4 inhibitor (TLR4-C34) in high glucose-induced inflammatory condition (Figure S3). These results imply that the inhibition of TLRs may indicate the anti-inflammatory effects of these sulfur compounds in the high glucose-induced inflammation.

### 2.2. Downregulation of the Canonical NF-κB Pathway by Sulfur Compounds

**Figure 2:**
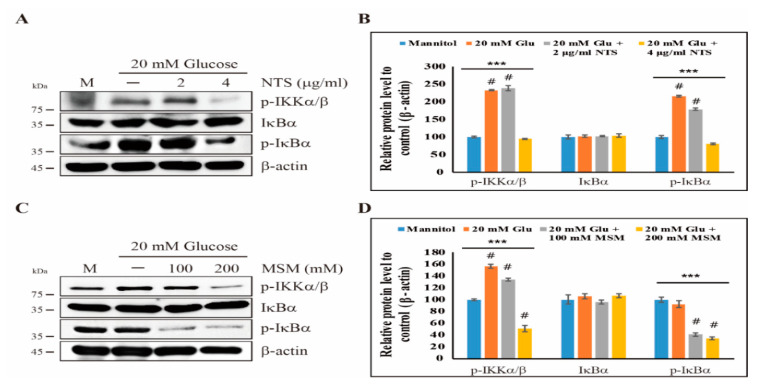
Sulfur compounds inhibit the canonical NF-κB pathway. (**A**) Western blotting analysis of THP-1 cells treated with 2 and 4 µg/mL NTS for 72 h showing the inhibition of high glucose-induced p-IKKα/β, IκBα, and p-IκBα expression. (**B**) The representative expression levels of proteins following NTS treatment were determined by densitometry and normalized to β-actin. The values are presented as means ± SEM of three independent experiments performed in duplicate (*n*  =  3). *** *p* < 0.001 (*ANOVA* test). # The mean difference is significant at the 0.001 level. (**C**) The expression levels of p-IKKα/β, IκBα, and p-IκBα proteins in THP-1 cells were detected by Western blotting after treatment with 20 mM glucose and MSM (100 and 200 mM) for 72 h. (**D**) The representative expression levels of proteins following MSM treatment were determined by densitometry and normalized to β-actin. The values are presented as means ± SEM of three independent experiments performed in duplicate (*n*  =  3). *** *p* < 0.001 (*ANOVA* test). # The mean difference is significant at the 0.001 level.

We observed the inhibition of inflammatory receptors TLR2 and TLR4 by NTS and MSM. Therefore, we next determined the effects of sulfur compounds on the canonical NF-κB pathway, including IκBα, IKKα, and IKKβ, as a downstream of TLRs. The results showed that 20 mM glucose induced the expression of phosphorylated IκBα and IKKα/β in THP-1 cells. However, 4 μg/mL NTS decreased the phosphorylation of high glucose-induced IκBα and IKKα/β (Figure 2A,B). MSM (200 mM) also showed similar results as NTS (Figure 2C,D). Specifically, MSM inhibited the high glucose-induced expression of proteins in the canonical NF-κB pathway. Therefore, these results suggest that the anti-inflammatory effects of these sulfur compounds might be mediated through NF-κB pathway.

### 2.3. Sulfur Compounds Downregulates ROS-Dependent PKC-Mediated Upstream Targets of NF-κB

**Figure 3:**
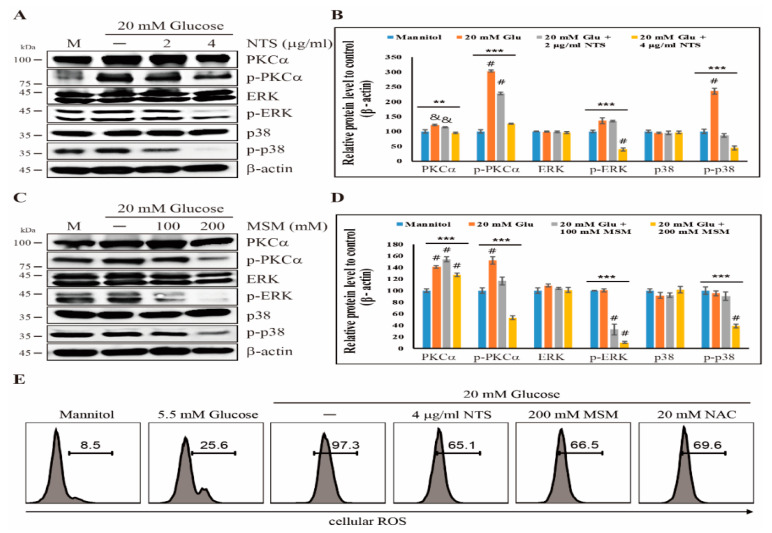
Sulfur compounds inhibit the PKC pathway. (**A**) Western blotting analysis of THP-1 cells treated with 2 and 4 µg/mL NTS for 72 h showing the inhibition of high glucose-induced p-PKCα, p-ERK, and p-p38 expression. (**B**) The representative expression levels of proteins following NTS treatment were determined by densitometry and normalized to β-actin. The values are presented as means ± SEM of three independent experiments performed in duplicate (*n*  =  3). **p < 0.01 and *** *p* < 0.001 (*ANOVA* test). & The mean difference is significant at the 0.01 level. # The mean difference is significant at the 0.001 level. (**C**) The expression levels of high glucose-induced p-PKCα, p-ERK, and p-p38 proteins in THP-1 cells after treatment with MSM (100 and 200 mM) for 72 h were detected by western blotting. (**D**) The representative expression levels of proteins by MSM were determined by densitometry and normalized to β-actin. The values are presented as means  ±  SEM of three independent experiments performed in duplicate (*n* = 3). *** *p* < 0.001 (*ANOVA* test). # The mean difference is significant at the 0.001 level. (**E**) Flow cytometry analysis showing the inhibition of the high glucose-induced ROS by 4 µg/mL of NTS and 200 mM of MSM. 20 mM NAC (N-Acetyl-l-cysteine) was used as a positive control for antioxidant effects of sulfur compounds.

Sulfur compounds showed an inhibition of the canonical NF-κB pathway. Next, we determined the role of the PKC-dependent anti-inflammatory effects of NTS and MSM. We analyzed the expression patterns of PKCα, ERK, and p38 pathway in THP-1 cells treated with or without NTS or MSM for 72 h. Our results showed an increase in the phosphorylation of PKCα, ERK, and p38 with 20 mM glucose, which was decreased by 4 μg/mL NTS or 200 mM MSM (Figure 3A,C). The total forms of these proteins remained unchanged, indicating that sulfur compounds block only the activation of inflammatory molecules (Figure 3B,D). The inhibition of the upstream targets of NF-κB (PKC, ERK, and p38) suggests an anti-inflammatory effect of these sulfur compounds. We also studied the production of ROS in response to glucose and the inhibitory effect of sulfur compounds in ROS production by flow cytometry. ROS were highly upregulated in a 20 mM glucose condition and downregulated by 4 μg/mL and 200 mM MSM (Figure 3E). These results suggest that the inhibitory effects exerted by sulfur compounds MSM and NTS on PKC-mediated NF-κB activation may also take place through the downregulation of glucose-stimulated ROS production.

### 2.4. Sulfur Compounds Induce Anti-Inflammation Effects by Inhibiting NF-κB and its Proinflammatory Cytokines

**Figure 4:**
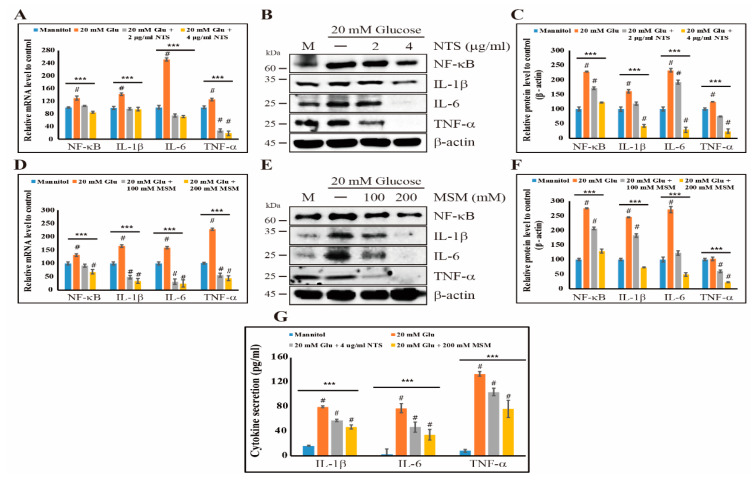
Sulfur compounds inhibit NF-κB and proinflammatory cytokines. (**A**) The mRNA expression levels of NF-κB, IL-1β, IL-6, and TNF-α in THP-1 cells were detected by real-time PCR after treatment with 20 mM glucose and NTS (2 and 4 μg/mL) for 24 h and normalized to GAPDH mRNA. The values are presented as means ± SEM of three independent experiments performed in duplicate (*n*  =  3). *** *p* < 0.001 (*ANOVA* test). # The mean difference is significant at the 0.001 level. (**B**) Western blotting analysis of THP-1 cells treated with 2 and 4 µg/mL NTS for 72 h showing the inhibition of the high glucose-induced expression of NF-κB, IL-1β, IL-6, and TNF-α. (**C**) The representative expression levels of proteins after NTS treatment were determined by densitometry and normalized to β-actin. The values are presented as means ± SEM of three independent experiments performed in duplicate (*n*  =  3). *** *p* < 0.001 (*ANOVA* test). # The mean difference is significant at the 0.001 level. (**D**) Real-time PCR mRNA data after treatment with 20 mM glucose and MSM (100 and 200 mM) for 24 h showing the relative expression levels of NF-κB, IL-1β, IL-6, and TNF-α normalized to GAPDH mRNA. The values are presented as means ± SEM of three independent experiments performed in duplicate (*n*  =  3). *** *p* < 0.001 (*ANOVA* test). # The mean difference is significant at the 0.001 level. (**E**) Western blotting analysis of THP-1 cells treated with 100 and 200 mM of MSM for 72 h showing the inhibition of high glucose-induced NF-κB, IL-1β, IL-6, and TNF-α expression. (**F**) The representative expression levels of proteins following MSM treatment were determined by densitometry and normalized to β-actin. The values are presented as means  ±  SEM of three independent experiments performed in duplicate (*n*  =  3). *** *p* < 0.001 (*ANOVA* test). # The mean difference is significant at the 0.001 level. (**G**) ELISA analysis showing the inhibition of the glucose-induced expression of NF-κB, IL-1β, IL-6, and TNF-α by 4 µg/mL of NTS and 200 mM of MSM. Mannitol was used as a negative control for high glucose-mediated cytokine expressions. The values are presented as means ± SEM of three independent experiments performed in duplicate (*n*  =  3). *** *p* < 0.001 (ANOVA test). # The mean difference is significant at the 0.001 level.

We found that sulfur compounds exhibit anti-inflammatory effects by modulating the canonical NF-κB pathway and inhibiting PKC/ERK/p38 signaling. Therefore, we next determined the effect of NTS and MSM on high glucose-induced NF-κB expression. Our results clearly supported our hypothesis. We observed that both NTS and MSM inhibited glucose-induced NF-κB expression at the mRNA level (Figure 4A,D). We also examined the expression of proinflammatory cytokines, including IL-1β, IL-6, and TNF-α (Figure 4B,E), and the results suggested an inhibition of the expression of these proinflammatory cytokines with 4 μg/mL NTS or 200 mM MSM (Figure 4C,F). These results provided more evidence of the anti-inflammatory effects of sulfur compounds. Then, we determined the effect of these sulfur compounds on the levels of NF-κB using an enzyme-linked immunosorbent assay (ELISA). The results showed an increase in the levels of IL-1β, IL-6, and TNF-α with 20 mM glucose, and 4 μg/mL NTS or 200 mM MSM decreased the levels of these cytokines (Figure 4G). These results also suggested the high glucose-induced anti-inflammatory effects of NTS and MSM.

### 2.5. Inhibition of NF-κB and Its Binding to Proinflammatory Cytokines by Sulfur Compounds

**Figure 5:**
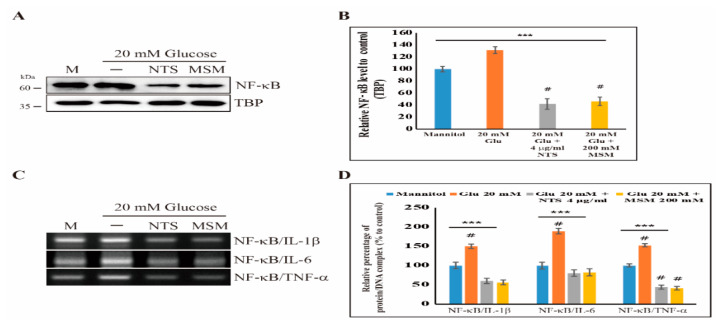
Sulfur compounds inhibit the nuclear translocation of NF-κB and its binding to proinflammatory cytokines. (**A**) Western blotting analysis of nuclear extracts harvested from THP-1 cells treated with 4 µg/mL NTS or 200 mM MSM for 72 h showing the inhibition of the high glucose-induced nuclear translocation of NF-κB. TBP (TATA-binding protein) was detected for nuclear loading control. (**B**) The representative expression levels of proteins after NTS treatment were determined by densitometry and normalized to TBP. The values are presented as means ± SEM of three independent experiments performed in duplicate (*n*  =  3). *** *p* < 0.001 (ANOVA test). # The mean difference is significant at the 0.001 level. *** *p* < 0.001 (*ANOVA* test). # The mean difference is significant at the 0.001 level. (**C**) ChIP assay analysis showing an inhibition of the binding of NF-κB protein to the DNA of IL-1β, IL-6, and TNF-α by treatment with 4 µg/mL NTS or 200 mM MSM for 72 h. (**D**) The representative expression levels of complexes following NTS and MSM treatment were determined by densitometry, and controls were set to 100%. The values are presented as means ± SEM of three independent experiments performed in duplicate (*n*  =  3). *** *p* < 0.001 (*ANOVA* test). # The mean difference is significant at the 0.001 level.

**Figure 6:**
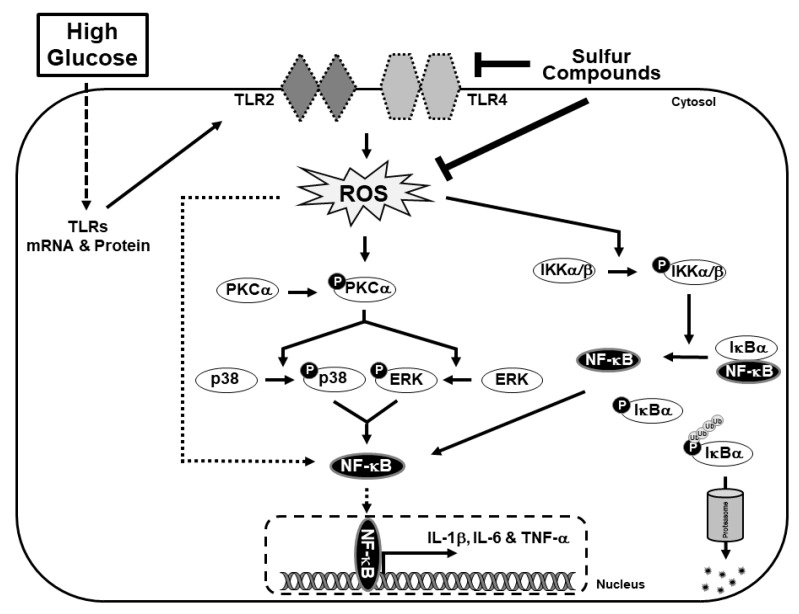
Molecular regulatory mechanism of high glucose-induced inflammatory responses by TLRs/NF-κB through ROS-mediated canonical and PKC-mediated pathways. The anti-inflammatory activities of NTS and MSM are mediated by inhibiting these pathways and the binding of NF-κB to proinflammatory cytokines.

From the Western blot analysis of NF-κB and its proinflammatory cytokines, we showed an anti-inflammatory effect. In order to further demonstrate this, we analyzed the translocation of NF-κB from the cytoplasm to the nucleus. The results suggested that treatment with 20 mM of glucose promoted the nuclear translocation of NF-κB, which indicates inflammation in response to high glucose (Figure 5A). However, treatment with NTS or MSM inhibited the translocation of NF-κB, which indicated an anti-inflammatory effect of these compounds (Figure 5B). Then, we examined the binding of NF-κB protein to the DNA of its proinflammatory cytokines, including IL-1β, IL-6, and TNF-α (Figure 5C). Using a chromatin immunoprecipitation (ChIP) assay, we confirmed that NTS and MSM inhibited the binding of NF-κB to its proinflammatory cytokines (Figure 5D). These results demonstrated the anti-inflammatory effects of NTS and MSM. Finally, our results suggest that these sulfur compounds exhibit anti-inflammation activity by regulating TLRs and NF-κB through ROS-mediated canonical and PKC/ERK/p38 pathways, as well as IL-1β, IL-6, and TNF-α signaling (Figure 6). Therefore, NTS and MSM may be drug candidates for the treatment of high glucose-induced inflammation. 

## 3. Discussion

The treatment of inflammation with natural substances is a promising approach because several natural compounds have high anti-inflammatory ability and less adverse effects [38]. An excess of glucose often leads to type 1 and type 2 diabetes, which are caused by inflammation. Sometimes, this results in the development of other associated diseases, including cancer [39,40]. Natural sulfur compounds, such as NTS and MSM, may be used as anti-inflammatory drugs if they can reduce high glucose-induced inflammation. Therefore, we hypothesized that NTS and MSM inhibit high glucose-induced inflammation by regulating molecular signaling pathways.

TLR2 and TLR4 are considered the central regulatory receptors of NF-κB-mediated inflammation in response to high glucose [41]. We found that 20 mM glucose increased the expression of TLR2 and TLR4 in THP-1 human monocytes, which indicated that inflammation was induced by high glucose. Therefore, if a drug can reduce or inhibit high glucose-induced TLR2 and TLR4 expression, it can be considered for its anti-inflammatory action. The results we obtained also supported our hypothesis. Specifically, NTS and MSM inhibited the high glucose-induced expression of TLR2 and TLR4 at both the transcriptional and translational levels. Flow cytometry analysis of TLR2 and TLR4 in THP-1 cells also confirmed the inhibition of the high glucose-induced expression of these receptors by NTS or MSM. From these preliminary results, we found that NTS and MSM may have the ability to induce anti-inflammatory effects by regulating NF-κB signaling. We also examined the levels of NF-κB in the presence of high glucose with or without NTS or MSM and found an increase in the expression of NF-κB, which was then decreased by NTS or MSM in THP-1 cells. This demonstrated that these sulfur compounds have the ability to reduce high glucose-induced inflammation by regulating TLRs and NF-κB signaling. Here, NF-κB plays a key role in the molecular signaling of sulfur compounds in high glucose-induced inflammation.

Canonical NF-κB signaling is a major pathway that is involved in inflammatory responses under hyperglycemic conditions [7]. The activation of the canonical NF-κB pathway through TLRs leads to the activation of inflammatory cytokines and the IKK complex [25]. Using Western blotting, we found that NTS and MSM inhibited canonical NF-κB signaling by downregulating the expression of phosphorylated IKKα/β and IκBα. Inflammatory responses through the IKK complex lead to the activation of NF-κB. Sulfur compounds reduced the expression of TLRs, the IKK complex, and NF-κB, which indicated that their anti-inflammatory effects are mediated through this molecular pathway. These results clearly indicate the anti-inflammation ability of NTS and MSM under high glucose conditions.

Under hyperglycemic conditions, NF-κB is activated by inflammatory cytokines in the canonical pathway. At the same time, the PKC pathway also mediates various cellular signals to activate NF-κB during inflammation [42]. TLR2 and TLR4 were closely associated with PKC signaling activation, and an increase in PKC activity was observed under diabetic conditions, indicating the upregulation of ROS [43]. A study showed that inflammation due to hyperglycemia leads to the activation of PKCα, which then activates NF-κB by inducing p38 and ERK1/2 to release IL-1β [25]. We also analyzed the anti-inflammatory ability of NTS and MSM in the PKC pathway. These results showed an increase in the expression of phosphorylated PKCα, p38, and ERK by high glucose, but their non-activated forms remained unchanged. In contrast, both NTS and MSM drastically downregulated these signaling molecules by downregulation of TLR expression and ROS production, demonstrating their anti-inflammatory ability. Therefore, both ROS-mediated canonical and PKC-dependent pathways activate NF-κB in response to inflammation, and sulfur compounds inhibited the activation of NF-κB. 

NF-κB is a key factor in high glucose-induced inflammatory response signaling. When NF-κB is activated in response to inflammation, it translocates from the cytosol to the nucleus and then acts as a transcription factor [44]. This leads to an increase in the expression of inflammation-associated genes, such as TNF-α, IL-1β, and IL-6 [45]. NTS and MSM inhibited NF-κB translocation and the expression of TNF-α, IL-1β, and IL-6. The inhibition of the expression of proinflammatory cytokines indicated the ability of sulfur compounds to induce anti-inflammatory effects. These sulfur compounds also inhibited the binding of NF-κB protein to the DNA of proinflammatory cytokines, including TNF-α, IL-1β, and IL-6, which supported the hypothesis that NF-κB plays a major role in the anti-inflammation ability of sulfur compounds. Using ELISA, we found that NTS or MSM reduced the high glucose-induced expression of proinflammatory cytokines, including TNF-α, IL-1β, and IL-6. Together, these results demonstrate the high glucose-induced anti-inflammatory ability of NTS and MSM through regulating NF-κB expression. In result, we demonstrated that the results of the biological tests with sulfur compounds indicate anti-inflammatory effects through the inhibition of high glucose-induced inflammatory pathway (Figure S4).

## 4. Materials and Methods

### 4.1. Antibodies and Reagents

The human monocyte cell line THP-1 was purchased from the Korean Cell Line Bank (Cancer Research Institute, Seoul National University, 28 Yongsan-dong, Chongno-gu, Seoul 110-744, Korea; 40202). Roswell Park Memorial Institute-1640 (RPMI-1640) culture medium, penicillin–streptomycin solution, trypsin-EDTA (0.05%), and CM-H2DCFDA (general oxidative stress indicator) (C6827) were purchased from Gibco (Thermo Fisher Scientific, Inc., 168 3rd Ave, Waltham, MA 02451, USA). D-glucose (G8270), D-mannitol (M4125), N-Acetyl-L-cysteine (A7250), TLR-IN-C34 (SML0832), and fetal bovine serum (FBS; 12003C) were purchased from Sigma-Aldrich (Merck KGaA, 3506 S Broadway, St. Louis, MO 63118, USA). A primary antibody specific for TLR2 (MBS850908) was obtained from MyBioSource (P.O. Box 153308, San Diego, CA 92195-3308, USA). Antibodies specific for TLR4 (sc-293072), p-IκBα (sc-8404), β-actin (sc-47778), and secondary antibodies (anti-mouse (sc-516102) and anti-rabbit (sc-2357)) were obtained from Santa Cruz Biotechnology, Inc. (10410 Finnell St, Dallas, TX 75220, USA). Primary antibodies specific for NF-κB (#8242), IκBα (#9242), p-IKKα/β (#9958), p-ERK (#9101), ERK (#9102), p-p38 (#4511), p38 (#8690), and IL-1β (#12703) were purchased from Cell Signaling Technology, Inc. (32 Tozer Rd, Beverly, MA 01915, USA). The PKCα (ab179523), p-PKCα (ab59411), IL-6 (ab6672), and TNF-α (ab183218) antibodies were obtained from Abcam (1 Kendall Sq B2304, Cambridge, MA 02139, USA). Methylsulfonylmethane (MSM) was purchased from OptiMSM^®^ (Bergstrom Nutrition; 1000 W 8th St, Vancouver, WA 98660, USA). Non-toxic sulfur (NTS) was made and provided by Nara Bio Co, 549-1 Yeoui-dong, Deokjin-gu, Jeonju, Jeollabuk-do, South Korea.

### 4.2. Cell Culture and Treatment

THP-1 cells were maintained in endotoxin-free RPMI-1640 (Gibco; 22400-089) supplemented with 50 μM mercaptoethanol, 10% FBS, 100 U/mL penicillin, and 100 μg/mL streptomycin at 37 °C and 5% CO_2_. Cells were cultured (1 × 10^6^ cells/mL) for 72 h (Western blotting and ELISA) or 24 h (real-time PCR) in either 20 mM glucose or 20 mM glucose containing the indicated concentrations of NTS or MSM. In addition, control cells were also cultured with 10 mM mannitol containing 5.5 mM glucose or only 5.5 mM glucose. Cell supernatants, lysates, and RNA were respectively harvested for ELISA, Western blotting, and real-time quantitative polymerase chain reaction (RT-qPCR).

### 4.3. Cell Vaibility Assay

THP-1 cells (1 × 10^4^ cells/100 μL) treated with various concentrations of NTS or MSM were seeded into 96-well plates and incubated for 72 h. After 72 h, the cultured cells were harvested, and the cells with 100 µL of medium containing CCK-8 (Dojindo, Kumamoto, JAPAN; the medium and CCK-8 volume ratio was 9:1) without FBS was added to each well of new plate. After incubation for 2 h at 37 °C in a CO_2_ incubator, the absorption at 450 nm of each well was measured using a microplate reader (BioTek, Winooski, VT, USA).

### 4.4. Real-Time qPCR

Total RNA was isolated with the RNeasy Mini Kit (Qiagen GmbH, Hilden, Germany) according to the manufacturer’s protocol. The extracted RNA was quantified spectrophotometrically at 260 nm, and cDNA was synthesized at 42 °C for 1 h and 95 °C for 5 min with a first-strand cDNA synthesis kit (K-2041; Bioneer Corporation, Daejeon, Korea) and oligo d(T) primers. Real-time qPCR was conducted in a thermal cycler (C1000 Thermal Cycler, Bio-Rad, Hercules, CA) as follows; 2 μL diluted cDNA was added to diluted forward and reverse primers (1 μL each, 100 pM) and 10 μL TB Green Advantage Premix (Takara Bio, Japan). The conditions used for the real-time qPCR were as follows; initial denaturation at 95 °C for 5 min, followed by 40 cycles of denaturation at 95 °C for 40 s, annealing at 58 °C for 40 s, extension at 72 °C for 40 s, and a final extension at 72 °C for 5 min. The primers used for the amplification are listed in Table S1. All measurements were performed in triplicate. The relative expression of the target genes was normalized to GAPDH. The calculations were performed using the Cp values obtained from the machine.

### 4.5. Western Blotting

Whole cell lysates were prepared from cells that were untreated or treated with glucose and NTS or MSM by incubating them on ice with a radioimmunoprecipitation lysis buffer (20–188; EMD Millipore) containing phosphatase and protease inhibitors to isolate protein. The protein concentrations were measured using the Bradford method (Thermo Fisher Scientific, Inc., Waltham, MA). Equal amounts of proteins (150 μg/well) were resolved by 10–15% SDS-PAGE. Then, the separated proteins were transferred onto nitrocellulose membranes. The blots were blocked for 1 h with 5% skim milk (BD Biosciences, CA; 90002-594) in TBS-T buffer (20 mM Tris–HCl (Sigma-Aldrich; Merck KGaA, St. Louis, MO; 10708976001), pH 7.6, 137 mM NaCl (Formedium, Norfolk, UK; NAC03), 0.1X Tween 20 (Scientific Sales, Inc. Oak Ridge, TN; 0777)) at room temperature. The membranes were then incubated with primary antibodies diluted in 5% skim milk overnight at 4 °C in a shaker. The membranes were then washed with TBS-T and incubated for 1 h with horseradish peroxidase-conjugated secondary antibodies at room temperature. Detection was conducted with a Femto Clean Enhanced Chemiluminescence Solution Kit (GenDEPOT; 77449; Katy TX), and images were acquired using a LAS-4000 imaging device (Fujifilm, Tokyo, Japan).

### 4.6. Flow Cytometry Analysis

After cultured cells were washed with pre-cold PBS, cell pellets were incubated with 10% BSA on ice for 20 min. Cells (3 × 10^6^) were stained with the following antibodies (1/200 diluted) on ice for 30 min; APC anti-human CD282 (TLR2) (Biolegend; 392304) and PE anti-human CD282 (TLR4) (Biolegend; 312806). Stained cells were washed with pre-cold PBS. Cytometry analysis was performed using a FACS Calibur flow cytometer (BD Bioscience). After the cultured cells were washed with prewarmed no-glucose glucose RPMI-1640 medium (GIBCO; 11879020), the cells were resuspended in 1 mL of staining buffer containing CM-H2DCFDA (5 μM) for cellular Reactive Oxygen Species (ROS). Then, the cells were incubated in a CO_2_ incubator at 37 °C for 30 min. The stained cells were washed with 1 mL of prewarmed PBS and used for FACS analysis. The analysis was performed using FlowJo software. 

### 4.7. ELISA

Cells were cultured (1 × 10^6^ cells/mL) for 72 h in 20 mM glucose containing the indicated concentrations of NTS or MSM. IL-1β, IL-6, and TNF-α were measured in the supernatants of cultured cells using a human ELISA kit (abcam; IL-1β: ab46052, IL-6: ab178013, and TNF-α: ab181421) according to the manufacturer’s instructions.

### 4.8. Nuclear Extraction Assay and NF-kB Detection

Cultured cells (1 × 10^6^ cells/mL) were treated with 20 mM glucose containing the indicated concentrations of NTS or MSM for 72 h, after being washed with pre-cold PBS. Nuclear protein extracts were prepared with the Nuclear Extract Kit (abcam 113474, Cambridge, MA, USA). Equal amounts of proteins (150 μg/well) were resolved by 10–15% SDS-PAGE. Then, the separated proteins were transferred onto nitrocellulose membranes. The blots were blocked for 1 h with 5% skim milk. The membranes were then incubated with NF-kB and TBP antibodies diluted in 5% skim milk overnight at 4 °C in a shaker. The membranes were then washed with TBS-T and incubated for 1 h with horseradish peroxidase-conjugated secondary antibodies at room temperature. Detection was conducted with a Femto Clean Enhanced Chemiluminescence Solution Kit (GenDEPOT; 77449; Katy TX, USA), and images were acquired using a LAS-4000 imaging device (Fujifilm, Tokyo, Japan).

### 4.9. ChIP Assay

The ChIP assay was performed using the Imprint ChIP kit (Sigma-Aldrich) according to the manufacturer’s protocol. Briefly, mouse muscle cells were fixed with 1% formaldehyde and quenched with 1.25 M glycine. After washing with PBS, the cells were suspended in a nuclear preparation buffer and shearing buffer and sonicated under optimized conditions. The sheared DNA was centrifuged, and the cleared supernatant was subjected to protein/DNA immunoprecipitation. The clarified supernatant was diluted with buffer (1:1 ratio), and 5 µL aliquots of the diluted samples were used as internal controls. Next, the diluted supernatant was incubated with anti-NF-κB antibodies in precoated wells for 90 min. The controls were incubated with normal goat IgG and anti-RNA polymerase II. Unbound DNA was removed using an immunoprecipitation wash buffer, and bound DNA was collected using the cross-link reversal method with DNA release buffer containing proteinase K. The released DNA and internal control DNA were purified using the GenElute Binding Column G. DNA was then quantified using conventional real-time PCR. The primer sequences were as follows. IL-1β, sense 5′-ATTGCCTCTTCCAGCAGCTT′ and antisense 5′-GGCTTCACTGAGGTTGCCTT-3′. TNF-α, sense 5′-TGGTGAGACAGAAAGAGCGG′ and antisense 5′-AGCCCTGAGGTGTCTGGT-3′. IL-6, sense 5′-GCTGATCCTGCCTCTGCC′ and antisense 5′-GACCCTCAAACCCACCCG-3′. The real-time PCR conditions were as follows; 95 °C for 5 min; 40 cycles of 95 °C for 30 s, 60 °C for 30 s and 72 °C for 40 s; and 72 °C for 5 min.

### 4.10. Statistical Analyses

The values are presented as means ± SEM of three independent experiments performed in duplicate (*n*  =  3). The statistical analyses were conducted via a one-way analysis of variance (ANOVA) with Tukey’s post-hoc test. The analyses were performed with the SAS 9.3 software program (SAS Institute, Inc., Cary, NC, USA). A *p*-value < 0.05 (*) was considered to indicate a statistically significant difference. # indicated the significance level.

## 5. Conclusions

In summary, natural sulfur compounds—NTS and MSM—inhibited high glucose-induced inflammation by regulating NF-κB expression in THP-1 human monocytes. The anti-inflammation activity of these sulfur compounds occurred through inflammatory response receptors, TLR2 and TLR4, and then by the inhibition of NF-κB activity through ROS-dependent canonical and PKC-dependent pathways to finally inhibit the proinflammatory cytokines TNF-α, IL-1β, and IL-6. From these results, we suggest NTS and MSM as drug candidates for anti-inflammation therapy.

## Supplementary Materials

The following are available online, Figure S1. Glucose induced the expression of proteins in the inflammation pathway. (A) Western blotting showing the effect of glucose treatment for 72 h on the expression of NF-κB, TLR4, p-IκBα, IκBα, p-IKKα/β, and ERK proteins in THP-1 cells. (B) The representative expression levels of proteins following treatment with increasing concentrations of glucose were determined by densitometry and normalized to β-actin. The values are presented as means ± SEM of three independent experiments performed in duplicate (*n* = 3). *** *p* < 0.001 (ANOVA test). # The mean difference is significant at the 0.001 level. Figure S2. Effects of NTS and MSM on viability in THP-1 cells. Cells were treated with various concentrations (NTS; 0–10 μg/mL or MSM; 0–500 mM) of two sulfur compounds for 72 h, and the cell viability was assessed by CCK-8 assay. The values are presented as means ± SEM of three independent experiments performed in duplicate (*n* = 3). *** *p* < 0.001 (ANOVA test). Figure S3. Sulfur compounds inhibited the expression of TLR4 and NF-κB. (A, B) Western blotting analysis of THP-1 cells treated with NTS and MSM for 72 h showing the inhibition of high glucose-induced NF-κB and TLR4 expression. TLR4-C34, a TLR4 inhibitor, was used for anti-inflammatory control in high glucose-induced inflammation. The values are presented as means ± SEM of three independent experiments performed in duplicate (*n* = 3). *** *p* < 0.001 (ANOVA test). # The mean difference is significant at the 0.001 level. Figure S4. Sulfur compounds inhibit the expression of inflammatory factors associated with high glucose-induced inflammation. **(A)** The mRNA expression levels of inflammatory factors in THP-1 cells were detected by real-time PCR after treatment with NTS (2 and 4 μg/mL) and MSM (100 and 200 mM) in 20 mM glucose for 24 h and normalized to GAPDH mRNA. Non-treated group (only 20 mM glucose) was used as a control to normalize or compare with NTS or MSM-treated groups. The values are presented as means ± SEM of three independent experiments performed in duplicate (*n* = 3). *** *p* < 0.001 (ANOVA test). # The mean difference is significant at the 0.001 level. (B) Graphical representation of western blotting analysis of THP-1 cells treated with NTS (2 and 4 μg/mL) and MSM (100 and 200 mM) for 72 h showing the inhibition of high glucose-induced inflammatory factors expressions. Non-treated group (only 20 mM glucose) was used as a control to normalize or compare with NTS or MSM-treated groups. The values are presented as means ± SEM of three independent experiments performed in duplicate (*n* = 3). *** *p* < 0.001 (ANOVA test). # The mean difference is significant at the 0.001 level.
